# Examination of the cut-off scores determined by the Ages and Stages Questionnaire in a population-based sample of 6 month-old Norwegian infants

**DOI:** 10.1186/1471-2431-11-117

**Published:** 2011-12-19

**Authors:** Astrid Alvik, Berit Grøholt

**Affiliations:** 1 Institute of Clinical Medicine, University of Oslo, Norway

## Abstract

**Background:**

Few population-based samples have previously published performance on the Ages and Stages Questionnaire (ASQ), a recommended screening tool to detect infant developmental delay. The aim of the study was to investigate performance on the ASQ in a population-based sample of 6-month-old infants.

**Methods:**

In this population-based questionnaire study from Oslo, Norway, the 30 item ASQ 6 month Questionnaire (N = 1053) were included, however without the pictograms, and compared to the Norwegian reference sample (N.ref) (N = 169) and to US cut-off values. Exclusion criteria were maternal non-Scandinavian ethnicity, infant age < 5.0 or > 7.0 months (corrected age), twins, and birth weight < 2.5 kg. Cut-off = 2.5 percentile (equivalent to mean minus 2 standard deviations). Pearson's Chi square and Mann-Whitney U were used to compare items and areas, respectively, with N.ref.

**Results:**

The reported ASQ scores were lower on all but one of the 10 significantly different items, and in all areas except Personal social, compared to the N.ref sample. The estimated cut-off values for suspected developmental delay (Communication 25, Gross motor 15, Fine motor 18, Problem solving 25 and Personal social 20) were lower than the recommended American (US) values in all areas, and lower than the Norwegian values in two areas. Scores indicating need for further assessment were reached by 13.8% or 20.5% of the infants (missing items scored according to the US or the Norwegian manual), and by 33.8% or 30.3% of the infants using the recommended US or the Norwegian cut-off values, in this population-based sample. The Fine motor area demonstrated a large variability depending on the different cut-off and scoring possibilities. Both among the items excluding pictograms and the items that do not have pictograms, approximately every third item differed significantly compared to the N.ref sample.

**Conclusion:**

The psychomotor developmental scores were lower than in the reference samples in this study of ASQ 6 month Questionnaire; to our knowledge the first study to be both representative and comparatively large. Approximately every third child with birth weight above 2.5 kg, received scores suggesting further assessment using recommended ASQ cut-off scores.

## Background

Early detection of infant developmental delay is important in order to gain early access to further assessment and intervention [1]. The American Academy of Pediatrics recommends that all infants and young children should be screened for developmental delays [1,2]. Further, use of specific screening tools has been shown to markedly increase the detection rate [3]. The validated American screening tool Ages and Stages Questionnaires (ASQ) [4] is recommended by the American Academy of Pediatrics for detection of developmental delay in infants and small children [2]. The ASQ is a set of 21 age-specific questionnaires intended for use from the age of 2 months to 5 1/2 years. Each questionnaire consists of 30 items (scoring "yes", "sometimes" or "not yet" depending on whether the child can perform the activity), covering five areas: Communication, Gross Motor, Fine Motor, Problem Solving, and Personal-Social. Children scoring at or below the cut-off on one or more areas should be considered for referral for further assessment.

The questionnaire may be used in a variety of settings (mail out, online, telephone interview, home visits and office of child care or physician) and both as parent report and report by health professionals [4]. Parent reports of child development are cost effective, and have become increasingly used over the past decades for screening and research purposes. The majority of parents have reported the ASQ as either very easy or easy to use, and not too time consuming [5,6]. Also, the ASQ has been reported to be the preferred screening instrument for developmental delay among pediatric residents [7], and the most commonly used instrument among community health care providers in parts of the US [8].

Few population-based studies have described performance on the ASQ in 6 month-old infants. For this age group, the American reference sample included 633 infants. Four hundred and ninety nine of these were infants of parents who had logged on to the ASQ web site and 134 were paper questionnaires completed by parents whose infants attended different programs for young children [4]. The Norwegian reference sample (N.ref) was a true random sample from the national population, including 169 infants at this age [9,10]. Thus, recommended cut-off values are determined based on either a non-randomized or a limited number of infants. Also, there is only limited reference data from Scandinavia and Europe concerning parents' responses on the ASQ. The US sample found no consistent pattern concerning web-based and paper questionnaires, and, therefore, combined the two methods [4]. However, little is known about whether alternative response formats (such as computer-administered versus paper based questionnaires, or presentation without pictograms) may influence parents' responses concerning the development of their child.

The aim of the present study was to report the results on the Ages and Stages 6 month Questionnaire in singleton infants with birth weight above 2.5 kg in a large population-based, ongoing longitudinal questionnaire study in Norway.

The aims were:

To describe the scores on the ASQ at 6 months of age, and to compare them with those obtained in a previously published Norwegian reference sample

To estimate cut-off levels for suspected developmental delay in the present sample, and to compare these levels with the cut-off levels in the American and the Norwegian reference samples.

To investigate whether there were indications of item differences due to the presence or absence of pictograms

## Methods

The data are part of a longitudinal, population-based questionnaire study. In Norway, all pregnant women attend free antenatal visits including a routine ultrasound screening at 17-18 weeks of pregnancy. In Oslo, approximately half of the population lives in the catchment area of Ulleval University Hospital. The pregnant women attending the ultrasound screening at Ulleval University Hospital are representative of pregnant women in Oslo. Women attending the screening between June 2000 and May 2001 were invited to join the study, ninety-two percent of whom accepted. Non-Scandinavian speaking and/or immigrants from non-western countries were not invited. The first questionnaire (T1 at 17 weeks of pregnancy) was filled out at the antenatal clinic. The questionnaires at T2 and T3 (at 30 weeks of pregnancy and six months after term) were sent by post to those returning the previous questionnaire. The questionnaires were completed by 1749 women at T1, 1424 women at T2 and 1303 women at T3. This constituted, at T1, 93% of those who joined and 86% of those invited to join the study, and at T2: 82% and at T3: 92% of those to whom the questionnaire was sent. The questionnaires received at T3 represented 75% of the initial cohort. For the present study, infants of mothers with non-Scandinavian ethnicity were excluded. Additional exclusion criteria were: twins, birth weight below 2.5 kg, and infant age < 5.0 months or > 7.0 months corrected age (= time after term). The sample had an N = 1053 after these exclusions. Birth data were collected from the Medical Birth Registry of Norway (MBRN). The data concerning the date of birth, collected from MBRN, were incomplete. Thus, premature infants were included, using corrected age, provided birth weight ≥ 2.5 kg. After exclusions according to the criteria, five infants registered as premature (from MBRN) were included (birth weight 2.7, 2.9, 3.0, 3.6 and 4.5 kg, hospitalized in the children's ward 10, 15, 0, 3 and 12 days, respectively).

The T3 questionnaire included the Norwegian translation of the Ages and Stages (ASQ) 6 month Questionnaire. The 30 items contains the response categories "Yes", "Sometimes" or "Not yet" concerning whether the child can perform the activity, with a respective score of 10, 5 or 0. The pictograms from the original ASQ were not included. The translation process of the Norwegian version of the ASQ was continued with some slight changes from the version received for use in this study and until publication. The minor changes introduced are expected, also by an independent expert, to have had no impact on the responses. The ASQ was scored according to the 2^nd ^US manual, i.e. one or two missing items in an area score were replaced by the ratio score of that area [11]. In analyses comparing with the N.ref sample, the dataset was scored according to the Norwegian manual, the difference being that in the latter, area scores not ending with 0 or 5 were rounded to the closest 0 or 5. An overall score was obtained by adding the five developmental area scores.

For maternal characteristics: age, education, income, civil status, and having older children, see Table 1.

**Table 1:** Maternal characteristics

	Mean (SD) or %
Maternal age^1^	30.8 (4.2)
Higher education^1^	66%
Higher income^1^	11.2%
Married or cohabitant^1^	97%
Older children	37%

^1^: Reported at pregnancy week 17-18, selecting the participants included in the present study

Higher education: Had qualified from, or studied at, the university or college

Higher income: More than NOK 400 000 (Norwegian crowns, equaling approximately 44400 USD or 30800 € at the time of T1)

All women provided written informed consent and permitted collection of data from the MBRN. The Regional Committee for Medical Research Ethics and the Norwegian Data Inspectorate approved the study.

### Statistical analyses

SPSS version 16 was used for all statistical analyses. ASQ Gross motor and Fine motor were reasonably normally distributed, while the other ASQ areas clearly had a skewed distribution (Skewness: Communication -.42; Problem solving -1.4; Personal social -.87; Standard error of skewness: .08 for all three). The 2.5 percentile, equivalent to mean minus 2 standard deviations (SD) used in the US reference study [4], were estimated for the areas. Comparing with the N.ref sample, Pearson's Chi square (2 degrees of freedom) were used comparing items, and Mann-Whitney U comparing the areas. In N.ref, the number in each items response category was calculated from the response percentage (published in the scoring sheet) and total N (N = 169 for all areas except Fine motor: N = 166) [9]. For the mean percentage area score, the number in each response category was added and divided by item number (for example "Not yet" was added for the 6 items/6) (Table 2).

**Table 2:** The mean percentage per area answering Not yet, Sometimes or Yes, and items significantly different from the Norwegian reference sample

	Dataset	N.ref	Items ≠ N.ref
	No/Somet/Yes	No/Somet/Yes	
**Communication**	12/29/59	10/24/66	2&5:***
**Gross motor**	33/11/56	28/10/62	4*;5***
**Fine motor**	22/22/56***	10/17/73	2,3,4&5:***
**Problem solv**.	7/18/76	5/14/81	1***;3*
**Personal soc**.	12/19/69	10/18/72	

N.ref: Norwegian reference sample

No/Somet/Yes: Not yet/Sometimes/Yes

Items ≠ N.ref: Items significantly different in the dataset and in the N.ref sample

Significance testing: Pearson's Chi Square (2 df), mean number in each category; for the N.ref sample, calculated from the percentage per category and the number of participants

* *p*-value < 0.05

** *p*-value < 0.01

*** *p*-value < 0.001

‡: 14 items have an ASQ Pictogram that is not included: Gross motor 4,5,6;Fine motor 3,4,5,6; Problem solving 4,5,6; Personal social 1,3,4,5

## Results

Participant characteristics are described in Table 1 and in the Method section. There were 51% boys in the sample.

In this study, the mean percentage per ASQ area to answer Not yet, Sometimes or Yes, are presented in Table 2. The mean percentage to answer Yes was lower in all areas, compared to the Norwegian reference sample (N.ref). Also, compared to the N.ref sample, 10 of 30 items differed significantly (Table 2). With the exception of one item (Communication 5), all differing items had lower values (lower infant performance) in the present study. At the area level, all areas except Personal social showed lower values of infant performance (*z *= -4.0, -3.8, -7.8 and -2.9 for Communication, Gross motor, Fine motor and Problem solving respectively; *p*-value < .001 for all except .003 for Problem solving) (Table 2). For comparison, the mean and SD per area is shown in Table 3, although most areas had a skewed distribution.

**Table 3:** Mean and standard deviation (SD) in the areas of Ages and Stages Questionnaire 6-months old

	Mean (SD)/N		Mean (SD)
	Dataset	N.ref	US ref N = 633
**Communication**	44.1 (9.5)/1048 ■	46.9 (9.2)/169	48.9 (9.6)
**Gross motor**	36.9 (11.7)/1052	40.4 (10.8)/169	45.6 (11.7)
**Fine motor**	40.1 (13.2)/1044	48.8 (10.4)/166	48.9 (11.9)
**Problem solv**.	50.8 (9.6)/1047 **■**	52.8 (8.3)/169	50.4 (11.4)
**Personal social**	47.2 (11.0)/1050 ■	48.7 (10.3)/169	48.3 (11.5)
**Total**	219.1 (36.4)/1035	237.8 (32.8)/166	-

American and Norwegian reference (N.ref) samples and the present dataset

**■**: Skewed distribution

The estimated cut-off levels (= 2.5 percentile) of the ASQ areas, shown in Table 4, were: Communication 25; Gross motor 15; Fine motor 18; Problem solving 25 and Personal social 20. According to the Norwegian manual [9], the area scores ending between 0 or 5, should be rounded to the closest 0 or 5. Applying this, the 2.5 percentile in the Fine motor area increased from 18 to 20. The percentage of infants scoring at or below the estimated cut-off levels was 13.8% (20.5% if Fine motor cut-off was rounded). Table 4 also presents the US and N.ref. cut-off levels [4,9] for comparison. Using the recommended Norwegian or US cut-off values, approximately every third infant scored at or below the cut-off in at least one area (30.3%/33.8%), most infants (73-80% depending on cut-off used) scoring positive in one area only.

**Table 4:** Cut-off scores in the present dataset and in the US and the Norwegian reference samples, and percentage of infants in the present dataset scoring at or below the various cut-off values

				Percentage scoring at or below		
	Dataset 2.5 perc/N	US Cut-off N = 633	**N.ref**. Cut-off/N	Dataset 2.5 perc	US Cut-off	**N.ref**. Cut-off
**Communication**	25/1048	29.65	25/169	4.6%	4.6%	4.6%
**Gross motor**	15/1052	22.25	15/169	5.3%	12.2%	5.3%
**Fine motor**	18/1044^1^ 20/1044^1^	25.14	25/166	18^1 ^= 2.6% 20^1 ^= 11.0%	21.2%	21.2%
**Problem solving**	25/1047	27.72	30/169	2.6%	2.6%	5.4%
**Personal social**	20/1050	25.34	20/169	3.0%	6.2%	3.0%

**Scoring at or below at least one area**				18^1 ^= 13.8% 20^1 ^= 20.5%	33.8%	30.3%

Recommended Cut-off scores in the US (Mean - 2SD) and Norwegian manual (primarily based on the 2 percentile) in the areas of Ages and Stages Questionnaire 6-months old, and 2.5 percentile in the present dataset (equivalent to a cut-off value at mean - 2SD, most areas had a skewed distribution)

^1^: The 2.5 percentile was 18 using the coding according to the US User guide 2, and 20 using coding according to the Norwegian manual (due to scores not ending on 0 or 5 to be rounded to the closest 0 or 5)

The Fine motor area demonstrated the greatest variability concerning the percentage of infants receiving a positive score, varying between 2.6% and 21.2%, depending on the chosen cut-off (Table 4). Excluding the Fine motor area, the number of infants achieving a positive score was, respectively, 12.8%, 19.7% or 14.8% using the 2.5 percentile, US or N.ref cut-off.

The 14 pictograms in the ASQ at 6 months were not included in the present study. Comparing these 14 items with the N.ref, five items had significantly lower values (Gross motor 4&5, and Fine motor 3,4&5)(Table 2).

The internal consistency measured by Cronbach's α was good for ASQ Total (.78), low for Communication (.36) and acceptable for the other areas (.53 - .65) (Table 5).

**Table 5:** The Internal consistency measured by Cronbach's α

	Dataset	**N.ref**.
**Communication**	.36	.45
**Gross motor**	.53	.52
**Fine motor**	.65	.58
**Problem solving**	.57	.65
**Personal social**	.53	.54
**Total**	.78	.79

N.ref: Norwegian reference sample

## Discussion

The main finding of this study of the Ages and Stages Questionnaire (ASQ) at 6 months was lower infant performance scores, than in the Norwegian, but also in the US reference samples. To our knowledge, this is the first study of ASQ at 6 months-of-age to be both representative and comparatively large.

The cut-off levels in the present sample were lower than the US levels in all areas. Compared to the Norwegian reference sample, N.ref, the cut-off levels were lower in the Fine motor and Problem solving areas. Approximately every third infant received a score indicating a need for further assessment using the recommended cut-off values. A lower percentage of infants scoring below the cut-off could be expected, as the study only included infants with birth weight above 2.5 kg. Further, the participating women were representative of pregnant women in Oslo, Norway, thus representing a population with little poverty.

There are two potentially important differences between the present sample and the N.ref sample. The present sample included 1053 infants, while N.ref, although containing questionnaires for several ages, included a relatively small sample of 6 months-old infants (N = 169). Thus, a cut-off at 2.5% would yield 4-5 infants below the cut-off in one area, as opposed to approximately 26 children in the present study. Further, both studies are population-based, but the present study is representative of the capital, while the N.ref study is representative of both urban and rural areas in Norway. Maternal age and education are not reported in the N.ref sample. In the present study, as expected in a population from the capital, there is a high percentage having higher education. Infants of mothers with high education have been found to score higher on the ASQ (i.e. better developmental scores) [12,13]. On the other hand, the maternal age is expected to be higher in the sample from the capital, and higher maternal age has been associated with lower infant performance [14].

The US study is not representative for a specific population. At 6 months of age, most responses were from parents who had logged on to the ASQ web site [4]. This could have introduced a selection bias, presumably in the direction of a higher infant performance compared to a representative sample. The authors found no consistent pattern of differences between the paper and web based responses across the age groups. Although mainly affecting sensitive questions, PC-based data collection methods have been shown to yield higher rates of unwanted behavioral outcomes, compared to self-administered questionnaires [15,16]. For parents, the development of your infant may be an emotional, although not exactly a sensitive, question. Potential response differences on the ASQ depending on administration format should be explored further.

It is important for infant health care to define cut-off values for suspected developmental delay that have sufficient sensitivity to ensure a high detection rate, but also sufficient specificity to avoid over referral. The fact that every third infant in this low risk population received a score indicating a need for further assessment using the recommended cut-off values, could be an indication of an unnecessarily high recommended referral rate, or poor specificity. In a community clinic study of 18 month old children, the ASQ had moderate sensitivity (0.67) but poor specificity (0.39) [5]. Other studies, however, have demonstrated that the referral rate of infants should be rather liberal. In one study, following 1363 term children not referred for or identified with delay, the referral rates were 5.6% or 8.1% according to pediatric or ASQ assessment respectively, at 12- or 24-month well-child visits [17]. In the 36-60 months follow-up, 20.8% received referrals of which 42.4% were eligible for services. For the 64 lower-risk predominantly late preterm children in the study, referral rates were 9.5% or 26.2% at 12- or 24 month, and at follow up it was 37.5% of which 50.0% were eligible for services [17]. Another study found infants with mild developmental delay, and those with false positive screening-results, to be an at-risk group which may benefit from further evaluation and intervention [18]. Further, mild developmental delay may be hard to detect [1]. The necessity of adequate cut-off levels in developmental screening instruments are further strengthened by a survey among US pediatricians providing health supervision to children up to 35 months-of-age [19]. The study showed that 65% reported inadequate training in developmental assessment. However, the finding in the present study that every third infant with birth weight above 2.5 kg in this low risk population needed further assessment, gives reason to question the recommended cut-off levels of ASQ for 6 month old infants.

At the area level, Fine motor had the most pronounced difference between the samples. The Fine motor area may be more susceptible to differences in cultures or subcultures than other areas, at 6 months of age. A study comparing ASQ in American and Korean children from 4 months to 5 years found differences between the results, particularly concerning the Fine motor area [20]. In the Korean study, including a limited number of 6 month old infants (N = 105), the Fine motor values resembled those in the present dataset (mean -2SD at 6 months of age: 17.65). The attitude in the culture towards infants using their fingers and palms to eat/play with the food, may affect the infant Fine motor score. Potential cultural differences may have uneven effect throughout infancy and childhood.

The results have varied when comparing ASQ samples from different cultures. One study, using ASQ 48 months, found mean population scores to be mostly similar when comparing Dutch results with US, Norwegian and Korean results [12]. In another study, the N.ref sample and the early US reference sample (in US User guide 2) [11] were compared [10]. This study, finding few differences at the area level, indicated that ASQ areas may be interpreted similarly in the two countries. The 10 age levels investigated in both samples were compared. However, when looking at age levels not included in the comparative study, the positive scores in the N.ref sample varied between 3% at 42 months-of-age to 38% at 18 months-of-age, using the US cut-off values [9]. At both these ages, the US cut-off values were deducted from the ASQ questionnaire at the age above and below [11]. The differences or similarities between these two samples could be based on inequalities in culture or representativity.

As the current study was part of an epidemiological study and had limited questionnaire space, the pictograms were not included. Further, the pictograms were expected to add little information to the short, direct form of the ASQ questions at 6-months-of-age, in this population with high reading abilities. Comparing the items where pictograms were excluded in the present dataset, with those in the N.ref sample, there were no systematic differences. Approximately every third item was significantly different whether a pictogram was excluded or not. In populations with little illiteracy, the ASQ at 6 months-of-age may function well without the pictograms. As these questions may be suitable for use in larger epidemiological studies which often have space limitations, this would be beneficial and should be explored further.

The analyses for internal consistency in each domain were generally comparable to the N.ref sample.

There are several strengths to the current study. First, the sample is population-based and relatively large (N = 1053). Also, the study has a relatively high response rate. Further, the Norwegian ASQ items are well translated and back-translated, and closely follow the original wording, thus there is little probability of translational distortion [9].

The study also has some limitations. First, the data to determine the gestational age were incomplete, and there is a possibility that some premature infants were not registered as such. Thus, our findings are valid for infants with birth weight ≥ 2.5 kg. Second, the small difference between the translation used in the study and the later published version, may represent a weakness, but probably had no impact on the responses. Further, although both the present sample and the N.ref sample are population-based, their representativeness differs somewhat and could potentially affect the comparison between the two. For instance, in the present dataset, maternal education was high. Education was not reported in the N.ref sample, but could be expected to be higher in a sample from the capital than in a sample representative for the entire population. However, if infants of mothers with high education score higher on ASQ as reported [12,13], this would strengthen the need for revised cut-off values.

## Conclusions

To our knowledge this is the first large and representative study of ASQ performance in 6-months-old infants. It demonstrates, in this low risk population, values of lower infant performance compared to the Norwegian, and also the US, reference samples. Using the recommended Norwegian or US cut-off levels, approximately every third infant with birth weight above 2.5 kg received scores indicating a need for further assessment. Using the 2.5 percentile of the study, equivalent to the US cut-off (Mean - 2SD), 13.8% of the infants received a positive score. This increased to 20.5% if missing items were scored according to the Norwegian, and not the US, manual. Adequate cut-off levels are important in screening instruments recommended for use in well child visits for all children.

There are indications that the ASQ 6 month questionnaire may function well without the pictograms in populations with mostly adequate reading abilities. This would be beneficial for epidemiological studies and should be explored further.

## Abbreviations

ASQ: Ages and Stages Questionnaires; MBRN: The Medical Birth Registry of Norway; N.ref: the Norwegian reference sample; SD: Standard deviation

## Competing interests

The authors declare that they have no competing interests.

## Authors' contributions

AA: Primary responsibility for the study design, data collection, analysis and interpretation, and in writing of the manuscript. BG: Participated in the analytic framework of the study, with the data interpretation, and in writing of the manuscript. Both authors read and approved the final manuscript.

## Pre-publication history

The pre-publication history for this paper can be accessed here:

http://www.biomedcentral.com/1471-2431/11/117/prepub
